# Patients Related Outcome Measures in Hand Surgery: What Perspective for Children’s Assessment?

**DOI:** 10.5704/MOJ.2303.024

**Published:** 2023-03

**Authors:** G Taccardo, D Vigliarolo

**Affiliations:** 1 Clinica Ortopedica, Universita Cattolica del Sacro Cuore, Milan, Italy; 2 Ageing, Neurosciences, Head-Neck and Orthopaedics Sciences, Fondazione Policlinico Universitario Agostino Gemelli IRCCS, Roma, Italy

Dear Sir,

The triennial IFSSH, IFSHT and FESSH combined congress of Hand Surgery has just been closed in London in June 2022. During the many sessions attended by surgeons and physical therapists, it became increasingly clear the need to use dedicated questionnaires for patients’ assessment. Although Disabilities of the Arm, Shoulder and Hand (DASH) Score and Patient-Reported Outcome Measurement System (PROMS) are also found to be adequate, Patient-Rated Wrist Evaluation (PRWE) and Michigan Hand Questionnaire (MHQ) for the assessment of outcomes are more appropriate for hand and/or wrist assessment^1,2^. Validation and cross-cultural adaptation of each individual questionnaire is long and investigative; however it is mandatory to have high-quality scientific studies, and to make reliable clinical assessments of patients in everyday clinical practice^1^.

Our recent experiences and current evidence suggest that there are no reliable PROMSs in the assessment of paediatric upper limb function, especially on content validity^2-4^. We need to look for new PROMSs-like instruments, in order to improve the quality of our work. Given the recent creation of the Paediatric and Adolescent Shoulder and Elbow Survey (Pedi-ASES)^5^, we believe there is a need for a study group or consensus conference to create a questionnaire/scorecard with child-specific activity assessment like as a CHILD-PRWE or a CHILD-MICHIGAN.

So far, we have to refer to objective clinical measurements and radiographic measurements. Actually, we also wondered whether we should create a new assessment tool that integrates objective and subjective assessments in the same score. We also inquire as to what age group we can consider suitable for use the PROMs we classically use for adults, and whether some kind of adjustments of the questions is needed to adapt them to different paediatric and adolescent age groups.
